# DRAGON-Data: a platform and protocol for integrating genomic and phenotypic data across large psychiatric cohorts

**DOI:** 10.1192/bjo.2022.636

**Published:** 2023-02-08

**Authors:** Amy J. Lynham, Sarah Knott, Jack F. G. Underwood, Leon Hubbard, Sharifah S. Agha, Jonathan I. Bisson, Marianne B. M. van den Bree, Samuel J. R. A. Chawner, Nicholas Craddock, Michael O'Donovan, Ian R. Jones, George Kirov, Kate Langley, Joanna Martin, Frances Rice, Neil P. Roberts, Anita Thapar, Richard Anney, Michael J. Owen, Jeremy Hall, Antonio F. Pardiñas, James T. R. Walters

**Affiliations:** MRC Centre for Neuropsychiatric Genetics and Genomics, Division of Psychological Medicine and Clinical Neurosciences, School of Medicine, Cardiff University, UK; MRC Centre for Neuropsychiatric Genetics and Genomics, Division of Psychological Medicine and Clinical Neurosciences, School of Medicine, Cardiff University, UK; and School of Psychology, Cardiff University, UK; MRC Centre for Neuropsychiatric Genetics and Genomics, Division of Psychological Medicine and Clinical Neurosciences, School of Medicine, Cardiff University, UK; and Directorate of Psychology and Psychological Therapies, Cardiff & Vale University Health Board, UK

**Keywords:** Genetics, schizophrenia, developmental disorders, bipolar affective disorders, attention-deficit hyperactivity disorders

## Abstract

**Background:**

Current psychiatric diagnoses, although heritable, have not been clearly mapped onto distinct underlying pathogenic processes. The same symptoms often occur in multiple disorders, and a substantial proportion of both genetic and environmental risk factors are shared across disorders. However, the relationship between shared symptoms and shared genetic liability is still poorly understood.

**Aims:**

Well-characterised, cross-disorder samples are needed to investigate this matter, but few currently exist. Our aim is to develop procedures to purposely curate and aggregate genotypic and phenotypic data in psychiatric research.

**Method:**

As part of the Cardiff MRC Mental Health Data Pathfinder initiative, we have curated and harmonised phenotypic and genetic information from 15 studies to create a new data repository, DRAGON-Data. To date, DRAGON-Data includes over 45 000 individuals: adults and children with neurodevelopmental or psychiatric diagnoses, affected probands within collected families and individuals who carry a known neurodevelopmental risk copy number variant.

**Results:**

We have processed the available phenotype information to derive core variables that can be reliably analysed across groups. In addition, all data-sets with genotype information have undergone rigorous quality control, imputation, copy number variant calling and polygenic score generation.

**Conclusions:**

DRAGON-Data combines genetic and non-genetic information, and is available as a resource for research across traditional psychiatric diagnostic categories. Algorithms and pipelines used for data harmonisation are currently publicly available for the scientific community, and an appropriate data-sharing protocol will be developed as part of ongoing projects (DATAMIND) in partnership with Health Data Research UK.

The value of collaboration and data-sharing is well recognised within the medical community and is one of the hallmarks of what has been called ‘the fourth age of research’, in which the pace of discovery has accelerated and international platforms for studying multifactorial problems have been built.^1^ The aggregation of data from individual research groups not only maximises the utility of individual data-sets and minimises demands on participants, but enables the joint analyses of complex data that can lead to incremental advances in elucidating disease aetiology.^2^ Within major psychiatric and neurodevelopmental conditions, few truly novel pharmacological treatments have been developed for several decades, with the noteworthy exceptions of ketamine for depression^3^ and atomoxetine for attention-deficit hyperactivity disorder (ADHD).^4^ Worryingly, many major pharmaceutical companies are decreasing their research efforts and investment in this area.^5^ This apparent stagnation in progress is the result of a lack of understanding of the pathogenesis of these conditions,^6^ hindering the identification of novel targets for drug discovery and limiting the utility of current diagnostic categories in defining mechanistically discrete disorders. A route to address these limitations involves integrating biological data at scale and across, rather than within, diagnostic classifications.^7^ Research conducted in this manner can explore the aetiological and biological commonalities between diagnoses revealed by genetic studies,^8^ accelerating discoveries on complex disorders and informing novel pharmacological and non-pharmacological therapeutic strategies, firmly grounded in biology.^9^

Recent large-scale studies have built on the hypothesis that psychiatric phenotypes do not always reflect distinct underlying pathogenic processes, and that some genetic risk factors are shared between neuropsychiatric disorders.^10^ This echoes the widely acknowledged clinical observation that many symptoms are features of multiple disorders and that patients often challenge current diagnostic classifications by presenting with characteristics of more than one disorder.^11^ What is currently not known, however, is to what extent this distribution of cross-disorder symptoms is related to the shared genetic liability between neurodevelopmental conditions.^10^ Commonalities in genetic risk factors might help identify a shared underlying biology, but this line of inquiry cannot be pursued without well-characterised cross-disorder samples, scarce even within large international consortia. In fact, it has been explicitly suggested that the majority of samples used in published genetic discovery studies have not been collected with the required amount of phenotypic data necessary to advance diagnostics, stratification and treatment.^12^ Thus, many research groups have directed their efforts to access resources with large amounts of routinely collected data, such as population biobanks and electronic health record systems, from which rich phenotypic data can be derived.^12,13^ However, some common limitations of these include selection biases and a low representation of clinically severe disorders.^13,14^ The latter can be exemplified by a recent study of schizophrenia genetic liability on 106 160 patients across four healthcare systems in the USA, where only 522 individuals with a formal diagnosis of schizophrenia were included;^15^ a small figure, but in line with a lifetime morbid risk of 0.7% for this disorder.^16^ Such is a classic quandary in psychiatric genomics,^17^ in which the setup of research studies leads to either a large case sample with minimal phenotyping or an extensively phenotyped one with fewer individuals.

## Aims and objectives

The Digital Repository for Amalgamating Genomic and Neuropsychiatric Data (DRAGON-Data) was therefore established at Cardiff University as a means of developing a platform where cross-disorder analyses of large well-phenotyped samples are possible. This approach integrates multiple existing case data-sets with genetic, clinical, environmental and developmental data. The focus on mental health across disorder boundaries and at scale aims to improve understanding of the pathophysiology of adult- and child-onset neurodevelopmental and psychiatric disorders, providing opportunities to combine diagnosis-led and symptom-led research. DRAGON-Data shares a focus with ongoing efforts to collate phenotype data within the Psychiatric Genomics Consortium (PGC),^18^ as well as previous mental health-related initiatives such as the Genetics of Endophenotypes of Neurofunction to Understand Schizophrenia (GENUS) Consortium,^19^ the International Consortium for Schizotypy Research (ICSR),^20^ the International 22q11.2 Deletion Syndrome Brain Behaviour Consortium (22q11.2DS IBBC),^21^ the Psychosis Endophenotypes International Consortium^22^ and the Genes to Mental Health (G2MH) Network.^23^ However, most of these projects have typically focused on a single psychiatric disorder or group of closely related conditions, whereas DRAGON-Data seeks to integrate genomic and phenotype data from a range of disorders across the developmental continua.

The current paper describes the formation of DRAGON-Data through the curation and harmonisation of phenotypic and genetic information across existing cohorts. These represent a broad diversity of psychiatric diagnoses, including ADHD, bipolar disorder, mood disorders, major depressive disorder, neurodevelopmental conditions, post-traumatic stress disorder and schizophrenia. This process has been informed by a series of legal and ethical considerations on the evolving landscape of individual-level data-sharing, which is required to ensure the sustainability of this repository as a resource for current and future researchers. Therefore, the governance framework of DRAGON-Data is also described, which enables the access and reuse of its data in ways that align with confidentiality regulations and the ethics of participating studies.

## Method

### Studies included

Fifteen studies from the MRC Centre for Neuropsychiatric Genetics and Genomics at Cardiff University (https://www.cardiff.ac.uk/mrc-centre-neuropsychiatric-genetics-genomics) were included in this project. A summary of the studies can be found in Table 1. Each study had its own approved research ethics, and ethical approval for the curation and development of DRAGON-Data was obtained from Cardiff University's School of Medicine Research Ethics Committee (approval reference 19/72). The studies included participants who were adults with psychiatric disorders, children (defined as up to age 18 years) with neurodevelopmental disorders, children of parents with psychiatric disorders and both children and adult carriers of rare neurodevelopmental risk copy number variants (ND-CNVs).

**Table 1:** Studies included in DRAGON-Data

Study	Reference	Main diagnosis	Principal investigator(s)	Genotyping platform	Number genotyped (after quality control)	Psychiatric instruments used	Diagnostic criteria included	Number phenotyped (harmonised)
BDRN	^24^	Bipolar disorder	N. Craddock, I. Jones, L. Jones	Affymetrix5 OmniExpress PsychChip	4806 8035 1102	SCAN	ICD-10, DSM-IV	6000
Bulgarian Trios								
Case–control data	^25^	Psychosis and mood disorders	G. Kirov	OmniExpress	806	SCAN	DSM-IV	305
Family data^a^	^26^	Probands with psychosis and mood disorders and their families	G. Kirov	Affymetrix6	2119	SCAN	DSM-IV	3084
CLOZUK	^27,28^	Treatment-resistant schizophrenia	J. T. R. Walters, M. Owen, M. O'Donovan	OmniExpress	13 743	None (anonymised samples)	None (anonymised samples)	16 405
Cardiff COGS	^29^	Schizophrenia, psychosis or bipolar disorder	J. T. R. Walters, M. Owen	OmniExpress	997	SCAN	ICD-10, DSM-IV	1301
DEFINE	^30^	Confirmed ND-CNV carrier	J. Hall, D. Linden, M.B.M. van den Bree, M. Owen	PsychChip	971 (number inclusive of ECHO and IMAGINE)	SCID PAS-ADD	DSM-IV	125
ECHO IMAGINE	^31,32^	Confirmed ND-CNV carrier	M.B.M. van den Bree, J.Hall, D. Linden, M. Owen	PsychChip		CAPA	DSM-IV	963
EPAD^a^	^33^	Major depressive disorder (at least one affected parent and their child)	F. Rice, A. Thapar	PsychChip	615	CAPA and SCAN	DSM-IV	674
F-Series^a^	^34^	Psychosis and mood disorders	M. Owen	OmniExpress	749	SCAN	ICD-10, DSM-IV	1022
DeCC/DeNt	^35^	Major depressive disorder	N. Craddock, L. Jones, C. Lewis, M. Owen	610 Quad	1346	SCAN	DSM-IV	1504
NCMH	^36^	Any developmental or mental disorder	I. Jones (and others)	PsychChip	3352	SCAN (*N* = 465) CAPS-5 PAS-ADD	For those with SCAN interviews: ICD-10, DSM-IV, DSM-5	16 311
PTSD Registry	^37^	PTSD	J. Bisson, N. Roberts	PsychChip	325	SCID CAPS	DSM-5	325
SAGE^a^	^38^	ADHD	A. Thapar, M. O'Donovan, M.J. Owen, K. Langley, J. Martin	HumanHap550 PsychChip	2073^a^	CAPA	ICD-10, DSM-IV	1132
Sib-Pairs	^39^	Schizophrenia	M. Owen	OmniExpress	918	SCAN	ICD-10, DSM-IV	918

BDRN, Bipolar Disorder Research Network; SCAN, Schedules for Clinical Assessment in Neuropsychiatry; COGS, Cardiff Cognition in Schizophrenia; DEFINE, Defining Endophenotypes From Integrated Neurosciences; ND-CNV, Neurodevelopmental Copy Number Variant; SCID, Structured Clinical Interview for DSM-IV; PAS-ADD, The Psychiatric Assessment Schedule for Adult with Developmental Disability; ECHO, Experiences of Children with copy number variants; IMAGINE, Intellectual Disability and Mental Health: Assessing Genomic Impact on Neurodevelopment; CAPA, Child and Adolescent Psychiatric Assessment; EPAD, Early Prediction of Adolescent Depression; DeCC/DeNt, Depression Case Control / Depression Network; NCMH, National Centre for Mental Health; CAPS-5, Clinician Administered PTSD Scale for DSM-5; PTSD, post-traumatic stress disorder; SAGE, Study of ADHD, Genes and Environment; ADHD, attention-deficit hyperactivity disorder.

a. Includes family data and/or (trios).

### Ethics approval

The development of DRAGON-Data was reviewed by the Cardiff University School of Medicine Ethics Committee as part of the ‘Clinical, phenotypic and genomic research in psychiatry’ application (reference SMREC 19/72), approved on 05/09/19. Ethical clearances to conduct each of the DRAGON-Data studies are detailed in their parent publications.

### Phenotypic data harmonisation strategy

**Fig. 1:**
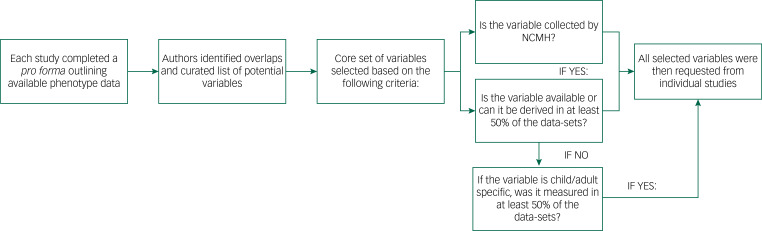
DRAGON-Data pipeline for phenotypic data curation. NCMH, National Centre for Mental Health.

The process of curating the phenotypic data is outlined in Figure 1, and a description of challenges we faced in our exercise is provided in Supplementary Appendix 1 available at https://doi.org/10.1192/bjo.2022.636. Initially, investigators from all studies completed a *pro forma* detailing the data and types of measures available, including the study clinical interviews, rating scales and self-report questionnaires. We compared all of the variables to identify overlaps and resolve situations where the same information might have been differently labelled across studies. We also defined a core set of variables (Table 2), focused on information relevant and applicable to cross-disorder research. A primary consideration for including a variable among this core set was whether it was collected as part of the National Centre for Mental Health (NCMH) research programme. The NCMH is a Welsh Government-funded research centre that investigates neurodevelopmental, psychiatric and neurodegenerative disorders across the lifespan. Its cohort is the largest sample with phenotype data available to us, and a cross-disorder resource in itself.^36^ As the NCMH is still being expanded by recruitment of participants, maximising its compatibility with DRAGON-Data was desired. Additionally, every core variable was required to be available in at least half of the current data-sets, taking into consideration that some data might be specific to child or adult cohorts. Variables that were not available in the NCMH and were present in fewer than half of the studies were only included if they could be derived from existing data to achieve the representation threshold. On receipt of each data-set, the variables were cleaned and matched with our defined core set of variables, and these were then signposted within our DRAGON-Data dictionary.

**Table 2:** List of phenotypic variables included in DRAGON-Data

Variables included	Number of studies	Number of participants
Symptoms		
Depression	12	15 410
Mania	11	13 906
Psychosis	9	12 072
ADHD	4	2460
Anxiety	4	2478
Conduct disorders	4	2460
Autism	4	2460
PTSD	1	325
Treatment history	13	31 164
Clinical/illness history		
Age at onset	10	29 023
Hospital admissions	7	26 372
Suicidal ideation	12	15 410
Adverse life events	6	9594
Education	9	24 790
Substance use	13	29 997
Family history of psychiatric illness	8	21 473
Physical health	11	27 725
Functioning		
Standardised measure of functioning (e.g. Global Assessment Scale)	5	6260
Marital/relationship status	7	23 290
Current occupation	7	25 597
Cognitive function	7	5048

Number of participants refers to the number of data points available for each set of variables listed. PTSD, post-traumatic stress disorder; ADHD, attention-deficit hyperactivity disorder.

### Genetic data harmonisation strategy

**Fig. 2:**
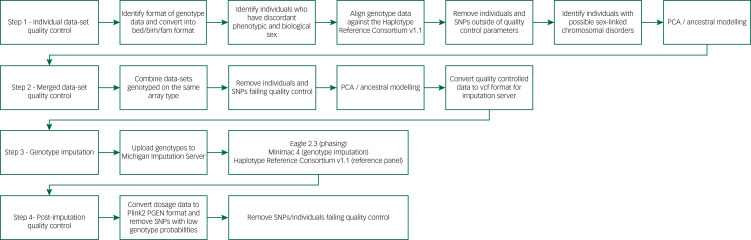
DRAGON-Data pipeline for SNP genotype quality control and imputation. PCA, principal component analysis; SNP, single nucleotide polymorphism.

We developed an in-house genotype quality control pipeline to facilitate standardised procedures for all aspects of genetic analysis (Fig. 2), available at https://github.com/CardiffMRCPathfinder/GenotypeQCtoHRC. The pipeline begins with conversion of genotype data into binary PLINK format.^40,41^ Genotyping platform, when not properly recorded in study logs, was inferred by comparing chromosome and base-pair positions of the genotypes on each data-set and 166 array manifests.^42^ Across the data-sets in DRAGON-Data, Illumina chips are by far the most common (Table 1). Despite the standardisation inherent to genotype data-sets that is driven by platform commonalities and the PLINK format conversion, creating a harmonised multi-study data-set requires stringent study-wide and data-set-wide quality control. We minutely describe these quality control steps and the challenges they are meant to address in Supplementary Appendix 1.

## Results

### The DRAGON-Data harmonised data-set

Table 2 displays an overview of the variables held by each study included in the final DRAGON-Data data freeze. A full list of the variables included in DRAGON-Data can be found in Supplementary Table 2, although the exact variables included varied between studies. All of the studies except for CLOZUK included a semi-structured clinical diagnostic interview, most commonly the Schedules for Clinical Assessment in Neuropsychiatry (SCAN^43^) for adults and the Child and Adolescent Psychiatric Assessment (CAPA^44^) for children and adolescents. Twelve of the fifteen studies collected data on individual symptoms. The NCMH study includes a brief assessment that does not include questions about individual symptoms, although a small subgroup of this sample (*n* = 485/16 311) completed more detailed interviews that included symptoms. The most common types of symptoms covered across all studies were depressive, manic and psychotic symptoms. Aside from symptoms, other variables with good coverage across studies were lifetime history of treatment (13/15), substance use (13/15) and history of suicidal ideation and attempts (12/15). The demographic characteristics of the studies are shown in Supplementary Table 1. The harmonised phenotype data is stored in a pseudonymised format within a secure database. There is an accompanying data dictionary cataloguing all available variables with names, descriptions and ratings, and cross-referencing comparable measures across the studies.

### Key recommendations for genotype-phenotype data harmonisation

Based on our experience developing DRAGON-Data, we suggest some recommendations for the harmonisation and analysis of clinical and genetic data:

- Consider the broad research questions that can be addressed with the creation of a clinical database. Consult with principal investigators and field researchers to identify the variables that will be needed to address these aims.
- Identify measures (e.g. questionnaires and interviews) that are in common across the data-sets included. These measures may be easier to harmonise for analysis, although factors outlined in Supplementary Appendix 1 (study protocol differences, use of diagnostic criteria) should be considered to ensure comparability.
- Record accurate information about each study variable, including measure used, version number, rating definitions, rating timeframe and source of information. This aids in the identification of comparable variables.
- Where new (secondary) variables have been derived by researchers and are designed to be comparable, information should be recorded about the (primary) variables used from each study to derive those secondary variables.
- A comprehensive data dictionary should accompany the database that incorporates the information outlined above. At a minimum, each variable should have recorded the name, description, values and corresponding labels (for categorical variables), as well as definition and coding of missing values. Within the data dictionary, variables should be highlighted if they are in common across the data-sets, as these may be suitable to analyse together. It is noteworthy that this curation and creation of dictionaries may often need to occur after the data collection, so researchers and funders should allow sufficient staff resources for the accurate completion of this task.
- Include basic demographic information to evaluate the representativeness of the sample, including age range, biological sex, gender identity, ethnicity and education.
- Data-sets do not need to be combined into a single data file. A database that houses the data-sets and allows an easy combination of selected studies and variables avoids the need for a single, large-scale data-set, and minimises the computational requirements for the querying and extraction of data.
- Data should only be shared and combined if there are suitable ethical and data-sharing agreements that participants have consented to. There may be separate ethical considerations for data-sharing within research settings and for linkage to other external data-sets, particularly public electronic health record databases.
- Imputation should only be performed on samples that have been genotyped on the same array backbone, or where there is substantial single nucleotide polymorphism (SNP) overlap after quality control. Furthermore, when performing quality control after imputation, removal of palindromic SNPs with high minor allele frequency (>0.4) is essential to minimising batch effects for samples genotyped on different arrays.
- When analysing copy number variants data across arrays, potential differences in probe density and coverage mean that it is vital that plots such as those for b-allele frequency drift, number of copy number variants called per individual and logR ratio standard deviation, are visually inspected to ensure the quality of the resulting calls.

## Discussion

### Using DRAGON-Data

All of the DRAGON-Data data have been securely stored in HAWK, a high-performance computing cluster supported by the Supercomputing Wales infrastructure,^45^ which comprises a network of 13 000 computer nodes distributed across four universities (Cardiff, Swansea, Bangor and Aberystwyth). This system allows the backed-up storage of genetic and phenotypic files, and their secure access by authorised users. Analysts in charge of curating genetic or phenotypic data are by default part of a ‘core project team’ with unrestricted access to the entire DRAGON-Data, whereas data-contributing researchers are granted access to their own raw and curated data for any purpose. Undertaking cross-disorder analyses is facilitated through a framework by which any curator or data-contributing researcher can send a structured analytic proposal to the board of investigators, who then decide whether to grant access to the relevant data on scientific grounds. This is modelled after successful international consortia such as the PGC,^18^ which in recent years has implemented responsible data-sharing practices among hundreds of investigators.

There are two main approaches to analysing the data within DRAGON-Data: combining individual-level information from across the studies (‘mega-analysis’) or through meta-analysis. Although the latter is relatively straightforward, jointly analysing all samples allows for a better assessment of heterogeneity in the data and can increase statistical power.^46^ However, combining samples is particularly problematic for the phenotypic data, as it requires recoding or modifying the variables to be comparable across studies, which could include deriving latent variables through factor analysis. Data combined in this way can be difficult to interpret because of the differences between studies outlined in the previous sections, and it is important to address this variability in both analytic techniques and interpretation of the results. Important considerations are whether the individual study variables are measuring the same construct and whether any variables derived from these are measuring the same construct as the original data. Note that none of these limitations applies to the genetic data, as (carefully) combining samples with large numbers of overlapping SNPs is a common procedure that is known to maximise both the number of successfully imputed variants and their quality.^47^ Thus, the suitability of a mega-analysis or meta-analysis approach for studies using DRAGON-Data will be decided based on the availability, characteristics and biases of the phenotypic data.

Outside of the data quality control pipelines, genetic analyses in DRAGON-Data can be undertaken using other consolidated tools, such as PLINK^40^ or GCTA.^48^ Responding to the rapid development of statistical methods to analyse complex phenotypes and ‘big data’, an effort has been made to integrate DRAGON-Data with the highly customisable R framework, via the use of data importers such as *GWASTools*^49^ and *bigsnpr.*^50^ This allows for use of the approximately 1700 tools currently offered by the Bioconductor suite^51^ in a large-scale genome-wide setting, and facilitates applying complex analytic techniques such as mixed-model regression^52^ and survival analysis.^53^ Large-scale genomic storage solutions have not currently been implemented in DRAGON-Data, as the weak compression implemented in PLINK files and related formats allows for efficient querying of genotype data even in its imputed form.^40,54^ However, these are active topics of research, and initiatives such as adopting the MPEG-G ISO standard will likely allow future data harmonisation projects to seamlessly incorporate whole-genome sequences.^55^

### Governance

For studies to be incorporated into DRAGON-Data, the lead principal investigator needed to confirm approval from their institutional ethics committee. The protection and confidentiality of participant data were of the utmost importance throughout the design of DRAGON-Data, and a number of safeguards were put in place to ensure the security, integrity, accuracy and privacy of participant data. First, in line with the required safeguards for processing special category data stipulated in the EU General Data Protection Regulation (GDPR; Article 89),^56^ the principle of data minimisation was respected, with only limited individual-level data being requested from research groups. Furthermore, as a means of maintaining the confidentiality and privacy of participants, all data were pseudonymised, and no personal or phenotypic information that allowed individuals to be re-identified was retained. As genome-wide genetic information cannot effectively be anonymised without compromising its integrity,^57^ all researchers accessing it must explicitly state that they will not attempt participant re-identification.

This project was conducted in line with Cardiff University's Research Integrity and Governance Code of Practice, and ethical approval for the curation and development of the DRAGON-Data was obtained from Cardiff University's School of Medicine Research Ethics Committee (reference 19/72). As described above, procedural safeguards were put in place to ensure secure, managed access to the data-set through the HAWK system, with the most privileges restricted to the ‘core analyst team’. In addition, a process of oversight has been implemented for the approval of secondary research proposals, which are reviewed by the lead principal investigator of each contributing sample, and must be approved before access to relevant requested data can be granted. All genetic analyses carried out by secondary investigators also have to be carried out within the HAWK environment, which allows their monitoring and auditing to rapidly detect data misuses.

### Challenges of data-sharing partnerships

The organisational challenges faced by DRAGON-Data highlight that potential data-sharing requirements should be considered, as much as reasonably possible, at the outset of any research study. Studies will benefit from having a data-sharing policy in place before the collection of any data, as a means of maximising the value of collected data, increasing transparency and ensuring responsible future sharing of data. This will depend on sharing with whom, and for what purpose. Consent processes have changed dramatically over the past 30 years and historical studies will not all have explicit consent on the data-sharing practices that are more commonly included today.^58^ In certain situations, additional ethical permission may be required for data-sharing when the sample is historical and or individuals can no longer be contactable. Thus, data-sharing without that explicit permission can only occur within certain circumscribed situations.

When obtaining consent for future research, researchers should aim to be as inclusive as possible, and allow participants to provide their written informed consent for general areas of research activity. In the context of broad consent, we would also advise the implementation of an oversight mechanism for the approval of future research studies. Participants entrust researchers to make reasonable decisions regarding future research on their behalf, and the process of oversight adds further protection to participants, since not all future research uses can be predicted.

### Limitations

Although there is rich demographic and clinical data available on patient cohorts in DRAGON-Data, the data on those without mental health disorders (‘controls’ in experimental study designs) is comparatively smaller and less detailed. The majority of the controls in DRAGON-Data came from the NCMH (*n* = 3508), and completed a brief interview that included demographic information and screening for the presence of psychiatric disorders. Four of the remaining studies in DRAGON-Data also collected data on participants without psychiatric diagnoses, but these were recruited because there were an unaffected sibling of a proband (Sib-Pairs cohort) or were ND-CNV carriers (ECHO, IMAGINE and DEFINE cohorts). Although these samples might not be representative of a standard control population, given their ascertainment, they might still be relevant for future DRAGON-Data studies. For example, merged data-sets with affected, relatives of affected, and unaffected individuals have been used for research into the additivity of risk factors for neurodevelopmental traits and in the validation of polygenic score methods.^59^

All of the studies in DRAGON-Data predated the publication of the ICD-11, which may have implications for how findings obtained using the data translate to current clinical practice. However, DRAGON-Data includes variables covering individual symptoms, onset and duration of illness, episodes and illness course, and this data could be used to derive diagnoses according to the most recent diagnostic criteria (ICD-11 and DSM-5). There was variation across the studies in how biological sex at birth and gender identity was measured and recorded, and many studies did not include standardised questions to probe sex at birth or gender identity. This is a common problem in historical data-sets, and even recent census questions on sex and gender for social science research vary across countries.^60^ An advantage of DRAGON-Data is the inclusion of genetic data, meaning biological sex can be identified for most participants. In addition, the largest sample with phenotype data in DRAGON-Data, the NCMH, included questions for both sex at birth and gender identity.

Finally, there is limited ancestry diversity within DRAGON-Data, as all of the included samples were recruited in the UK and contained a majority of individuals with European ancestry. Therefore, findings from DRAGON-Data may not be generalisable to individuals from different populations, although some cohorts (e.g. CLOZUK) can contain as much as 20% of non-European individuals from different ancestries or admixed backgrounds.^61^

### Open data prospects

At present, DRAGON-Data has been designed as a way of maximising the present and future utility of data collected at the MRC Centre for Neuropsychiatric Genetics and Genomics at Cardiff University during the past 30 years. Given the complexity of the data, particularly the phenotypic portion, the first cross-disorder analyses of DRAGON-Data have been carried out by members of the core analytic team and the participating investigator groups. Results of these analyses will be shared through Cardiff University online data repositories and communicated through standard scientific channels such as peer-reviewed publications. Ultimately, through adapting the PGC open-science model^62^ and taking advantage of the data-sharing frameworks supported by Health Data Research UK, such as the DATAMIND Hub,^63^ the DRAGON-Data resource will be available for external investigators where individual study consent and ethics permit such data-sharing. This will ensure compliance with the permissions and ethics of individual studies, and will be based on the secondary analysis principles detailed in the Governance section.

## Data Availability

All data relevant to the study are included in the article. Data from individual studies are available from multiple repositories and open resources as described in their parent publications (Table 1). Code for the genomic data harmonisation pipelines is available in a GitHub repository (https://github.com/CardiffMRCPathfinder/).

## Supplementary material

For supplementary material accompanying this paper visit http://doi.org/10.1192/bjo.2022.636. click here to view supplementary material
