# Supplementary material for: DRAGON-Data: a platform and protocol for integrating genomic and phenotypic data across large psychiatric cohorts
Source: BJPsych Open. 2023 Feb 8;9(2):e32. doi: 10.1192/bjo.2022.636 (PMC9970169; doi:10.1192/bjo.2022.636)
Supplement: Supplementary file 1 [file S2056472422006366sup001.docx]

SUPPLEMENTARY NOTE

[Challenges of harmonising phenotype data 2](#_Toc107498531)

[Measuring and rating psychopathology 2](#_Toc107498532)

[Sampling from the Population 2](#_Toc107498533)

[Study Protocol Differences 3](#_Toc107498534)

[Diagnosis 3](#_Toc107498535)

[Challenges of harmonising genotype data 4](#_Toc107498536)

[Format and genome assembly standardisation 4](#_Toc107498537)

[Sex-based quality control 5](#_Toc107498538)

[Call-rate quality control 5](#_Toc107498539)

[Assessment of population structure 5](#_Toc107498540)

[Genotype imputation 6](#_Toc107498541)

[Copy Number Variant Calling 6](#_Toc107498542)

[Batch effects after accounting for different genotyping platforms 7](#_Toc107498543)

[Dataset-wide duplicate samples 7](#_Toc107498544)

[Processing of public GWAS summary statistics 7](#_Toc107498545)

[Supplementary Table 1 8](#_Toc107498546)

[Supplementary Figure 1 10](#_Toc107498547)

[Supplementary Figure 2 11](#_Toc107498548)

[Supplementary References 12](#_Toc107498549)

Challenges of harmonising phenotype data

Measuring and rating psychopathology

The individual studies that form DRAGON-Data were designed using standard protocols for psychiatric research, and collected similar phenotypic data. However, they also used a range of different interviews, rating scales and questionnaires. This creates well-known challenges for data harmonisation^1^. In general, it should be noted that caution has to be exercised when amalgamating data from different studies even when these claim to use the same measures. Potential differences can include:

- Versioning: Measures can differ considerably between versions, with items being added or removed and definitions changing.
- Rating definitions: Ordinal scales can be named (e.g. 1=“mild”, 2=“moderate”, etc) resulting in a categorical or integer variable depending on study protocol. Some scales (e.g. OPCRIT^2^) can include items for which decimal point rating is acceptable, which could be transformed into continuous variables.
- Rating timeframes: Symptom and event data can be evaluated over different timeframes spanning weeks, months or years; and recorded as current, worst or lifetime occurrences. When integrating adult and child studies, it should be considered that events defined for the “lifetime” are not directly comparable due to intrinsic differences in this period of assessment. Measures that evaluate personality and behavioural traits might also not be completely consistent given the changes in these throughout the lifetime^3^.
- Sources of information: A difference between adult and child studies is that the latter are more likely to use multiple informants (participants, their siblings, parents and teachers). Harmonising all these reports can be difficult and might also require a prior compatibility assessment^4^.

The considerations above apply to individual studies, but they can add particular difficulty to reflect complex outcomes in a larger harmonised dataset. As an example, we highlight the different ratings of suicidal ideation across the DRAGON-Data studies (**Supplementary Table 1**). Note that these studies differed in whether they considered single versus multiple suicide attempts, duration of suicidal ideation or seriousness of attempts. This is likely to reflect the existence of different definitions of suicidal behaviour used in different research contexts^5^, and illustrates one of the challenges that can be faced when merging data from different studies.

To mitigate these challenges, we created a record of the interviews or ratings scales used by each study, along with the time period covered (e.g. lifetime, current), to facilitate the identification of comparable variables for analysis. This information is available within the DRAGON-Data dictionary. We also ensured that the coding of variables was consistent across comparable measures. Where possible, we derived comparable measures from existing data. For example, if some studies had collected “number of months of antidepressant use” and others had collected “ever taken antidepressants”, we would use the data from the first set of studies to create an “ever taken antidepressants” variable thereby ensuring that consistent variables were available across all datasets. It should be noted that this approach can be reductive, as the finer detail in variables are lost in favour of binary measures. For this reason, we also retained the original variables in DRAGON-Data. Given the volume of data in DRAGON-Data, we intend to continue deriving variables on a needs basis, working with researchers using the datasets to build on the data repository and generate coding scripts to facilitate variable derivation and analysis.

Sampling from the Population

Recruitment strategies and inclusion criteria can affect the characteristics of the samples, creating differences between them and making them unrepresentative of the population from which they are drawn. It has been suggested that participants enrolled in research studies of serious mental illness display better functional outcomes than are typical for those with the disorders in the wider population^6^, when compared against naturalistic samples from outpatient services^7^. Population cohort studies also suggest that those with more severe psychopathology and higher genetic loading for psychiatric disorder are more likely to drop out, leading to under-representation particularly in longitudinal samples^8^. The media used to approach these participants can also play a role in the sample characteristics, with internet-based recruitment engaging larger proportions of ethnic minorities and highly-educated female individuals than traditional face-to-face settings^9^. For most studies in DRAGON-Data, recruitment was based on clinically ascertained, prevalent cases and therefore they are likely to have over-sampled participants with severe, chronic illness and under-sampled individuals who recovered and/or were discharged from services. Additionally, in common mental health conditions such as depression and anxiety, this might also over-represent women who are more likely to access help than affected males^10^. A special case in terms of sample composition also concerns the DEFINE, ECHO and IMAGINE studies, which specifically recruited carriers of ND-CNVs and represent a novel genetics-first approach whereby participants are ascertained based on known genetic risk rather than psychiatric phenomenology^11^. Including these samples might have important implications for research examining genotype-phenotype associations in the combined dataset, as improperly accounting for their genotype-led recruitment would bias calculations on the prevalence of genetic or environmental risk factors. However, it is important to integrate genetic and phenotype approached to enable comparative research into the role of these risk factors in people with and without highly penetrant genetic variants^12^.

Study Protocol Differences

Samples were recruited using a mix of longitudinal and cross-sectional designs. The existence of a follow-up period in longitudinal studies establishes a temporal order for symptom and event measures, which provides another level of detail over the broader definitions found in cross-sectional designs. The cross-sectional studies collected a mixture of current, worst episode and lifetime symptom measures. As it has been previously described in the context of causal inference^13^, it is not advisable to combine longitudinal measures into or with “lifetime ever” variables, since this assumes that the events they reflect did not occur outside of the study assessment periods. Other issues that can affect the compatibility of different designs are attrition (in longitudinal studies), participant issues in completing assessments (e.g. length of time required) and the mode in which the study was conducted. Within DRAGON-Data most studies were conducted face to face with participants but also utilised telephone interviews, postal questionnaires and online data collection. This could affect how questions are interpreted and in turn, the likelihood and content of participants’ responses. In addition, there is evidence that participants may be more willing to disclose sensitive information in interview or face to face settings than in others^14^.

An advantage of DRAGON-Data is that the studies were conducted within the same department, and many used similar protocols for sample recruitment and data collection. As part of our data curation, we recorded what measures each study had used and what time period was covered, allowing us to identify commonalities in protocols across the studies. This information was then incorporated in the DRAGON-Data dictionary. The data dictionary indicates which measures are comparable across the studies. For example, CardiffCOGS, F-Series, Sib-Pairs, Bulgarian Case-Control and Family studies, BDRN, DeCC/DeNt and NCMH (subsample) all used the Schedules for Clinical Assessment in Neuropsychiatry (SCAN) interview to collect lifetime clinical data. The studies also used similar recruitment protocols and focused on recruiting adults with a history of psychiatric diagnosis from secondary psychiatric services. This meant we were able to identify many variables across the studies that had been collected using standardised questions and were comparable. Therefore, the creation of DRAGON-Data can facilitate cross-disorder analysis of thousands of participants with psychosis, bipolar disorder and depression. Similarly, the studies of childhood and adolescent mental health used the same interview (Child and Adolescent Psychiatric Assessment, CAPA) and thus, we have comparable measures for children and adolescents with ADHD, those at risk of depression and those who are ND-CNV carriers.

Diagnosis

Due to the different focus of individual DRAGON-Data studies, there were differences in the ways that diagnoses were made. Most studies used standardised interviews and medical records (where available) to derive consensus research diagnoses, with CLOZUK validating their ascertainment (based on prescription of the antipsychotic clozapine) against research interviews^15^. The NCMH population sample used self-report, asking participants to report diagnoses that they had been given by a health professional. This is an approach taken by other large studies such as the UK Biobank^16^. While data obtained via self-reports can be of poorer resolution than that from a structured interview, this approach has the advantage of allowing faster recruitment of larger samples^17^. The accuracy of self-report diagnoses needs also to be considered, which may differ by diagnosis. Self-reported diagnoses of specific, chronic mental health conditions that require involvement with secondary psychiatric services, such as schizophrenia, may be more accurate than reports of common mental health conditions, such as depression, that typically encompass a wide range of presentations and can be diagnosed and treated in a variety of health settings. This can introduce variability in defining phenotypes with impacts on study results. Research attempting to estimate the heritability of depressive disorders using inconsistent diagnostic criteria classically demonstrated this^18^; and recent work employing samples with broad, self-report definitions of depression to identify genetic risk loci have also resulted in signals that are not specific to this condition^19^. To ameliorate these problems, the studies included in DRAGON-Data have focused on categorical diagnoses rated according to the Diagnostic and Statistical Manual of Mental Disorders (DSM) or International Statistical Classification of Diseases (ICD) criteria. The most commonly used criteria by the studies were DSM-IV followed by ICD-10, with some studies using both. We separated the diagnosis variables according to diagnostic criteria in our DRAGON-Data dictionary (see Table 1), as the same diagnosis across the two sets of criteria may not be comparable. This has been demonstrated in a study comparing ICD-10 and DSM-IV in a cross-disorder sample, which showed that the two diagnostic systems have high concordance for depression, substance dependence, generalised anxiety disorder but low concordance for post-traumatic stress disorder and substance harmful use or abuse^20^.

Another consideration is that all the studies predated the publication of ICD-11 and most predated the publication of DSM-5, which may impact how any findings using the data translate to current psychiatry practice. Whilst only small changes were made to some diagnostic criteria from ICD-10 to ICD-11, other diagnostic criteria received more substantial changes^21^. For example, the threshold for diagnosis of post-traumatic stress disorder (PTSD) was raised such that patients now need three core symptoms (re-experiencing the traumatic event, avoidance of thoughts/reminders of the event, persistent perception of heightened threat) to receive a diagnosis. This means that some of the research participants with a diagnosis of PTSD in DRAGON-Data may not meet the new threshold in ICD-11, and those who do are likely to have a more severe presentation^22^. An advantage of DRAGON-Data is the inclusion of data covering individual symptoms, onset and duration of illness, episodes and illness course. This data could be used to derive diagnoses according to ICD-11 and DSM-5 criteria.

While ICD and DSM are standard criteria, it has been proposed that a better approach to diagnostic classification may be to focus on dimensional measures of psychopathology, such as the National Institute for Mental Health’s Research Domain Criteria (RDoC^23^). This approach may be adopted in the future as it could facilitate combining datasets to conduct cross-disorder research, given that many symptoms overlap diagnostic boundaries, such as the overlapping mood and psychotic symptoms observed in both schizophrenia and bipolar disorder^24^. DRAGON-Data could facilitate research in this area given the shared symptom measures used across the studies, particularly amongst the studies examining mood and psychotic disorders.

Challenges of harmonising genotype data

Format and genome assembly standardisation

To maximise the number of SNPs available for imputation, we performed alignment of local genotype data against the Haplotype Reference Consortium (HRC) panel v.1.1^25^ using Genotype Harmoniser v1.42^26^. Genotype Harmoniser is a Java-based application that compares SNP information in the user data against a reference dataset such as an imputation panel. Where discordant SNP information is present, for example due to allele mismatches, strand flips or different SNP identifiers, the user genotype data is updated to match that of the reference panel. We have observed that differences in genome build between the original and reference dataset result in Genotype Harmoniser discarding large numbers (e.g. more than 50%) of the original SNPs. If present, instances of this behaviour are flagged by our pipeline and solved via a local implementation of the widely-used Liftover Tool^27^ to retrieve physical coordinates in the appropriate b37/hg19 format.

In DRAGON-Data, a variety of genotyping arrays were used both within and between studies. This presents challenges for merging and imputing datasets. All the genotyping arrays analysed have a large set of common variants (a “GWAS backbone”), with most differences due to the inclusion of custom markers tagging rare exonic variation. The accuracy of genotype imputation is improved with larger sample sizes, plateauing around 2,000 samples^28^, though there must also be sufficient numbers of genotyped markers (at least 200,000 SNPs^29^) that overlap with the imputation reference panel after genotype quality control. We, therefore, grouped datasets that were genotyped on the same, or similar arrays. This resulted in four separate imputation batches for samples genotyped on the OmniExpress, PsychChip/Illumina HumanCoreExome, Illumina 610 Quad/Illumina HumanHap550 and Affymetrix5 platforms.

Sex-based quality control

We performed checks for discordant phenotypic and biological sex using the “sex-check” function in PLINK v1.9. This function is reliant on the presence of at least one sex chromosome. Discordant findings in the absence of complementary information from the individual (e.g. a disclosure of gender transitioning) are suggestive of either a sample mix-up during genotyping or an inaccurately recorded phenotype. If no resolution can be reached these samples are excluded from further analysis. Where no sex information is present in the original dataset, the sample is retained. If genotype calls from both sex chromosomes are present, call rates at the Y chromosome are used to assess the presence of individuals with sex-linked chromosomal disorders such as Turner (X0) or Klinefelter (XXY) syndromes^30^. Individuals with suggestive sex-linked chromosomal disorders are flagged for further investigation.

Call-rate quality control

We removed SNPs with low call rates (<0.95), individuals with low genotyping rates (<0.95), markers that fail the Hardy-Weinberg Equilibrium test (mid-p<10^-6^) and those with a minor allele frequency (MAF) < 0.01. Only autosomal SNPs were retained. Duplicated individuals were removed unless they belong to known monozygotic twin pairs; however, first degree relatives are retained for studies with trio or family designs. This is the final step of the pre-imputation QC. Afterwards genotypes are converted to VCF format using PLINK, sorted using vcftools v0.116^31^ and compressed to .gz format.

Assessment of population structure

While not strictly part of a QC process, the generation of principal components (PCs) using genotype data is needed to identify and account for population and ancestral substructures that can bias the results of association studies^32^. Our pipeline addresses this by generating PCs using the GENESIS suite, implemented in R. Within it, the PC-AiR^33^ function allows us to process both unrelated and family-based datasets, as it accounts for known or cryptic relatedness via the calculation of genotype relatedness matrices (GRMs). PCs generated by this method can readily be used to correct for population structure in regression-based analyses.

A more detailed ancestry analysis is also performed on each dataset, following a similar procedure to that described in Legge et al. 2019^34^. First the available SNPs are restricted to those on the set of 167 ancestry informative markers (AIMs) contained in the EUROFORGEN^35^ and 55-AISNP^36^ forensic panels, many of which are common across the different Illumina genotyping platforms. Afterwards, the dataset is merged with a public reference panel with known ancestries, a combination of the Human Genome Diversity Project (HGDP)^37^ and South Asian Genome Project (SAGP)^38^ datasets. This reference contains 1108 samples from 62 worldwide populations, which have been subdivided in 7 biogeographical ancestries^39^ (“Subsaharan African”, “North African”, “European”, “Southwest Asian”, “East Asian”, “Native American” and “Oceanian”). In order to perform the ancestry inference, a number of PCs, determined using the Tracy-Widom test for eigenvalues^32^, are then derived solely on the reference panel, and a prediction model is trained using Fisher’s Linear Discriminant Analysis algorithm. The samples with unknown ancestries are then “projected” onto the reference panel PCs^40^, and their ancestry is estimated using the prediction model. At least 80% probability of a given ancestry is required to automatically assign an individual to it, though the admixture patterns of individuals not achieving this probability can still be manually examined.

Genotype imputation

The Michigan Imputation Server (MIS) is a cloud-based resource that facilitates haplotype pre-phasing and genotype imputation^41^. The MIS also houses the HRC panel, containing genotypes of over 60,000 individuals across multiple ancestral backgrounds^25^. There are substantial improvements in imputation quality using the HRC reference over 1000 genomes, particularly at lower MAF thresholds^42^. The MIS also performs some SNP quality control before phasing, including removal of SNPs if they contain irregular allele codes, duplicate IDs, indels, monomorphic SNPs, discordant alleles between the user and population reference panel alleles and low call rates of < 0.9. Though other options are available, our dataset is processed via Eagle v2.3 pre-phasing^43^ and MiniMac3 imputation^41^ using HRC v1.1 as the reference panel.

After genotype imputation, imputed data is stored in .vcf.gz format, with accompanying info files containing information about the quality of imputed variants. Data is converted into .pgen format using PLINK v2 and subsequently into standard .bed/.bim/.fam format. Specifically, we remove SNPs where individual genotype probabilities are < 0.9, MAF <1%, genotyping rate < 0.95 and HWE < 1E-4. SNPs can be extracted at various imputation quality thresholds (R^2^). A conversion to best-guess genotypes is also performed in PLINK v2, after applying imputation quality thresholds (INFO < 0.3).

Copy Number Variant Calling

Most of the samples in DRAGON-Data include raw genotype information, enabling us to perform copy number variant (CNV) calling. We developed an in-house CNV QC pipeline to facilitate standardised procedures for all aspects of this procedure (**Supplementary Figure 1**), available at <https://github.com/CardiffMRCPathfinder/NeurodevelopmentalCNVCalling>.

First, we extract b-allele frequencies and logR ratios for each sample using Illumina Genome-Studio v2.05. CNV calling is performed using PennCNV v1.05 with genomic control correction^44^. CNVs are subsequently merged if the total distance between CNVs is less than 50% of their combined length. Appropriate population frequency of the B allele (PFB) and guanine/cytosine (GC) content files are generated as recommended by PennCNV. Filters are applied to remove CNVs with fewer than 20 probes, less than 20KB in length or with confidence scores < 5. Individuals are excluded if they have more than 30 CNVs, large logR ratios > 0.35 or high or low wavefactor (less than -0.03 or greater than 0.03), however, for future uses, these parameters might have to be re-examined and modified depending on the genotyping platform used.

Initially, CNVs called using this pipeline are cross-referenced against a list of 54 pathogenic CNVs known to confer increased risk of schizophrenia, autism, intellectual disability and major depressive disorder^45^. There are several advantages to prioritising these CNVs: First, they are typically large (>100KB) and are more reliably called across different genotyping platforms. Second, these CNVs are pleiotropic and lack complete penetrance for specific disorders, meaning they are good candidates for investigating associations with psychiatric cross-disorder phenotypes.

Batch effects after accounting for different genotyping platforms

We observed substantial batch effects in the pairwise comparison of samples after undergoing routine QC. Further inspection of the data revealed this was caused by palindromic SNPs (AT/TA or CG/GC genotypes), which resulted in erroneous allele frequencies which differed across datasets when the minor allele frequency was high (> 0.4). This issue was only apparent after merging datasets, which mirrors the experience of the eMERGE consortium^46^. Removal of these SNPs resulted in the loss of obvious batch effects across the first 10 PCs tested.

Dataset-wide duplicate samples

It is not uncommon for the same individual to be recruited into more than one psychiatric research study. Unless the individual voluntarily reports they have participated in a known existing study, this information would not be known to researchers in other groups. We identified 1909/41957 duplicate individuals (4.5%) across the entire dataset using genetic relatedness checks as implemented in PLINKv2 and retained the sample with the highest number of high quality imputed markers.

Processing of public GWAS summary statistics

When performing genetic analyses such as polygenic risk scoring, LD score regression or other analyses, multiple GWAS summary statistics are required. Despite some proposals for standardisation*^47^*, the output from GWAS software is still highly variable and even lacks consistent headings across individual studies. Processing of these files is thus not user-friendly, typically requiring manual curation, for example filtering by imputation quality, allele frequency or changing header names to match the required format of specific programs. To address these issues, we developed an R pipeline (*summaRygwasqc*) that automatically processes GWAS summary statistics files and performs quality control filtering, aligns SNP information against the HRC reference panel and converts summary data to a standardised format that is compatible with PRSICE2*^48^* , PRScs*^49^* and LDSC*^50^* (**Supplementary Figure 2**). This code is available at <https://github.com/CardiffMRCPathfinder/summaRygwasqc>.

Supplementary Table 1

**Rating scales for suicidal ideation across the studies**

| Study | Suicidal Ideation: Rating Scale |
| --- | --- |
| CoMPaSS | 1. Absent 2. Tedium Vitae 3. Suicidal Ideation 4. Attempt unlikely to result in death 5. Attempt likely to result in death 6. Multiple attempts likely to result in death |
| NCMH | 1. Absent 2. Tedium vitae 3. Suicidal ideation 4. Attempt unlikely to result in death 5. Attempt likely to result in death 6. Multiple attempts unlikely to result in death 7. Multiple attempts likely to result in death |
| ECHO, IMAGINE, SAGE & EPAD (children only) | Binary variables (yes/no) covering:   - Thoughts about death or suicide - Suicide attempts - Non-suicidal self-harm |
| EPAD (parents only) | Suicide attempt or self-harm:   1. Mild 2. Moderate 3. Severe |
| PTSD Registry | Question covers suicide attempts and self-harm in the context of borderline personality disorder:   1. Inadequate information 2. False or absent 3. Sub-threshold 4. Threshold or true |
| Sib-Pairs & F-series | 1. None 2. 1 week duration or one attempt 3. 2 weeks duration 4. At least one month |
| Bulgarian Trios (family and case data) | 1. Not present 2. Thoughts but no attempts 3. Attempt at suicide 4. Serious attempt 5. Multiple serious attempt |
| BDRN | Suicidal ideation:   1. Yes 2. No 3. Unknown |
| DeCC/DeNt | 1. Deliberately considered but no attempt 2. Injured self or made attempt but no serious harm 3. Suicide attempt resulting in serious harm 4. Suicide attempt designed to result in death 5. Uncertain |

Note: No variable for suicidal ideation or attempts in DEFINE

Supplementary Figure 1


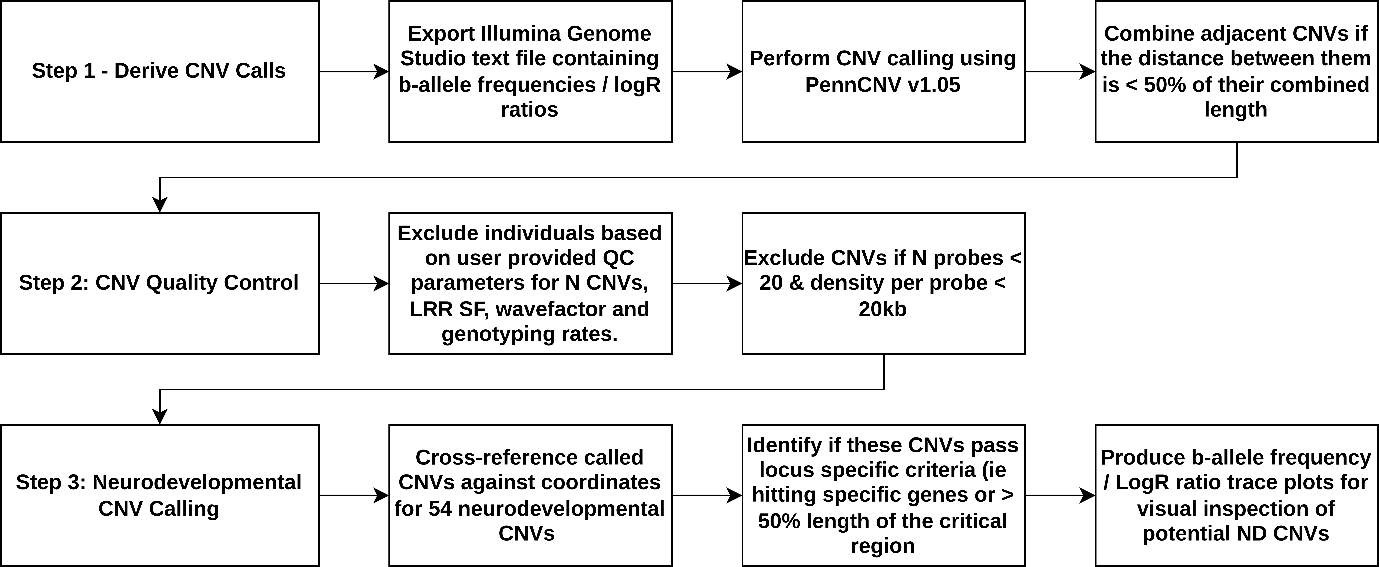


DRAGON-Data pipeline for CNV Calling.

Supplementary Figure 2

**
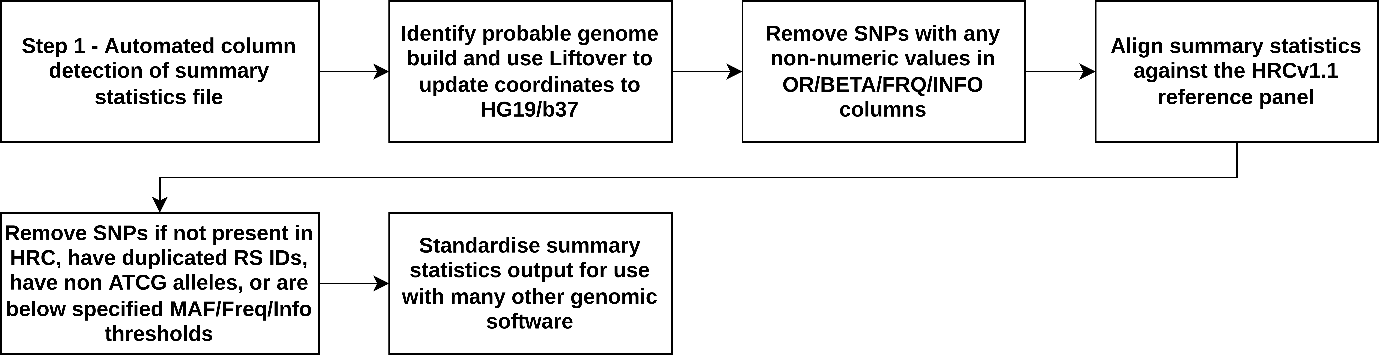
**

DRAGON-Data pipeline for standardising external genome-wide association study (GWAS) summary statistics.

Supplementary References

1. Bath PA, Deeg D, Poppelaars JAN. The harmonisation of longitudinal data: a case study using data from cohort studies in The Netherlands and the United Kingdom. *Ageing and Society* 2010;30(8):1419-37. doi: 10.1017/S0144686X1000070X [published Online First: 2010/09/29]

2. McGuffin P, Farmer A, Harvey I. A polydiagnostic application of operational criteria in studies of psychotic illness: development and reliability of the OPCRIT system. *Arch Gen Psychiatry* 1991;48(8):764-70.

3. Roberts BW, DelVecchio WF. The rank-order consistency of personality traits from childhood to old age: A quantitative review of longitudinal studies. *Psychol Bull* 2000;126(1):3-25. doi: 10.1037/0033-2909.126.1.3

4. Baldwin JS, Dadds MR. Reliability and Validity of Parent and Child Versions of the Multidimensional Anxiety Scale for Children in Community Samples. *J Am Acad Child Adolesc Psychiatry* 2007;46(2):252-60. doi: <https://doi.org/10.1097/01.chi.0000246065.93200.a1>

5. Klonsky ED, May AM, Saffer BY. Suicide, Suicide Attempts, and Suicidal Ideation. *Annual Review of Clinical Psychology* 2016;12(1):307-30. doi: 10.1146/annurev-clinpsy-021815-093204

6. Lally J, Watkins R, Nash S, et al. The Representativeness of Participants With Severe Mental Illness in a Psychosocial Clinical Trial. *Frontiers in Psychiatry* 2018;9(654) doi: 10.3389/fpsyt.2018.00654

7. Kline E, Hendel V, Friedman-Yakoobian M, et al. A comparison of neurocognition and functioning in first episode psychosis populations: do research samples reflect the real world? *Soc Psychiatry Psychiatr Epidemiol* 2019;54(3):291-301. doi: 10.1007/s00127-018-1631-x

8. Martin J, Tilling K, Hubbard L, et al. Association of Genetic Risk for Schizophrenia With Nonparticipation Over Time in a Population-Based Cohort Study. *Am J Epidemiol* 2016;183(12):1149-58. doi: 10.1093/aje/kww009

9. Batterham PJ. Recruitment of mental health survey participants using Internet advertising: content, characteristics and cost effectiveness. *International Journal of Methods in Psychiatric Research* 2014;23(2):184-91. doi: 10.1002/mpr.1421

10. Judd F, Komiti A, Jackson H. How Does Being Female Assist Help-Seeking for Mental Health Problems? *Aust N Z J Psychiatry* 2008;42(1):24-29. doi: 10.1080/00048670701732681

11. Arnett AB, Wang T, Eichler EE, et al. Reflections on the genetics-first approach to advancements in molecular genetic and neurobiological research on neurodevelopmental disorders. *Journal of Neurodevelopmental Disorders* 2021;13(1):24. doi: 10.1186/s11689-021-09371-4

12. Cleynen I, Engchuan W, Hestand MS, et al. Genetic contributors to risk of schizophrenia in the presence of a 22q11.2 deletion. *Mol Psychiatry* 2020 doi: 10.1038/s41380-020-0654-3

13. Wunsch G, Russo F, Mouchart M. Do We Necessarily Need Longitudinal Data to Infer Causal Relations? *BMS: Bulletin of Sociological Methodology / Bulletin de Méthodologie Sociologique* 2010(106):5-18.

14. Booth-Kewley S, Larson GE, Miyoshi DK. Social desirability effects on computerized and paper-and-pencil questionnaires. *Computers in Human Behavior* 2007;23(1):463-77. doi: 10.1016/j.chb.2004.10.020

15. Pardiñas AF, Holmans P, Pocklington AJ, et al. Common schizophrenia alleles are enriched in mutation-intolerant genes and in regions under strong background selection. *Nat Genet* 2018;50(3):381-89. doi: 10.1038/s41588-018-0059-2

16. Fry A, Littlejohns TJ, Sudlow C, et al. Comparison of Sociodemographic and Health-Related Characteristics of UK Biobank Participants With Those of the General Population. *Am J Epidemiol* 2017;186(9):1026-34. doi: 10.1093/aje/kwx246

17. Davis K, Hotopf M. Mental health phenotyping in UK Biobank. *Prog Neurol Psychiatry* 2019;23(1):4-7.

18. McGuffin P, Katz R. The Genetics of Depression and Manic-Depressive Disorder. *Br J Psychiatry* 1989;155(3):294-304. doi: 10.1192/bjp.155.3.294 [published Online First: 2018/01/02]

19. Cai N, Revez JA, Adams MJ, et al. Minimal phenotyping yields genome-wide association signals of low specificity for major depression. *Nat Genet* 2020;52(4):437-47. doi: 10.1038/s41588-020-0594-5

20. Andrews G, Slade T, Peters L. Classification in psychiatry: ICD–10 versus DSM–IV. *Br J Psychiatry* 1999;174(1):3-5. doi: 10.1192/bjp.174.1.3 [published Online First: 2018/01/02]

21. Gaebel W, Stricker J, Kerst A. Changes from ICD-10 to ICD-11 and future directions in psychiatric classification *Dialogues Clin Neurosci* 2020;22(1):7-15. doi: 10.31887/DCNS.2020.22.1/wgaebel [published Online First: 2020/07/24]

22. Barbano AC, van der Mei WF, Bryant RA, et al. Clinical implications of the proposed ICD-11 PTSD diagnostic criteria. *Psychol Med* 2019;49(3):483-90. doi: 10.1017/S0033291718001101 [published Online First: 2018/05/14]

23. Cuthbert BN, Insel TR. Toward the future of psychiatric diagnosis: the seven pillars of RDoC. *BMC medicine* 2013;11(1):126.

24. Owen Michael J. New Approaches to Psychiatric Diagnostic Classification. *Neuron* 2014;84(3):564-71. doi: <https://doi.org/10.1016/j.neuron.2014.10.028>

25. McCarthy S, Das S, Kretzschmar W, et al. A reference panel of 64,976 haplotypes for genotype imputation. *Nat Genet* 2016;48(10):1279-83. doi: 10.1038/ng.3643

26. Deelen P, Bonder MJ, van der Velde KJ, et al. Genotype harmonizer: automatic strand alignment and format conversion for genotype data integration. *BMC Research Notes* 2014;7(1):901. doi: 10.1186/1756-0500-7-901

27. Kuhn RM, Haussler D, Kent WJ. The UCSC genome browser and associated tools. *Briefings in Bioinformatics* 2012;14(2):144-61. doi: 10.1093/bib/bbs038

28. Stanaway IB, Hall TO, Rosenthal EA, et al. The eMERGE genotype set of 83,717 subjects imputed to ~40 million variants genome wide and association with the herpes zoster medical record phenotype. *Genet Epidemiol* 2019;43(1):63-81. doi: 10.1002/gepi.22167

29. Pistis G, Porcu E, Vrieze SI, et al. Rare variant genotype imputation with thousands of study-specific whole-genome sequences: implications for cost-effective study designs. *Eur J Hum Genet* 2014;23:975. doi: 10.1038/ejhg.2014.216

30. Igo Jr. RP, Cooke Bailey JN, Romm J, et al. Quality Control for the Illumina HumanExome BeadChip. *Current Protocols in Human Genetics* 2016;90(1):2.14.1-2.14.16. doi: 10.1002/cphg.15

31. Danecek P, Auton A, Abecasis G, et al. The variant call format and VCFtools. *Bioinformatics* 2011;27(15):2156-58. doi: 10.1093/bioinformatics/btr330

32. Patterson NJ, Price AL, Reich D. Population structure and eigenanalysis. *PLoS Genet* 2006;2(12):e190. doi: 10.1371/journal.pgen.0020190

33. Conomos MP, Miller MB, Thornton TA. Robust inference of population structure for ancestry prediction and correction of stratification in the presence of relatedness. *Genet Epidemiol* 2015;39(4):276-93.

34. Legge SE, Pardiñas AF, Helthuis M, et al. A genome-wide association study in individuals of African ancestry reveals the importance of the Duffy-null genotype in the assessment of clozapine-related neutropenia. *Mol Psychiatry* 2019;24(3):328-37. doi: 10.1038/s41380-018-0335-7

35. Phillips C, Parson W, Lundsberg B, et al. Building a forensic ancestry panel from the ground up: The EUROFORGEN Global AIM-SNP set. *Forensic Sci Int Genet* 2014;11:13-25. doi: 10.1016/j.fsigen.2014.02.012

36. Kidd KK, Speed WC, Pakstis AJ, et al. Progress toward an efficient panel of SNPs for ancestry inference. *Forensic Sci Int Genet* 2014;10:23-32. doi: 10.1016/j.fsigen.2014.01.002

37. Li JZ, Absher DM, Tang H, et al. Worldwide Human Relationships Inferred from Genome-Wide Patterns of Variation. *Science* 2008;319(5866):1100-04. doi: 10.1126/science.1153717

38. Chambers JC, Abbott J, Zhang W, et al. The South Asian Genome. *PLOS ONE* 2014;9(8):e102645. doi: 10.1371/journal.pone.0102645

39. Tishkoff SA, Kidd KK. Implications of biogeography of human populations for'race'and medicine. *Nat Genet* 2004;36:S21-S27.

40. Reich D, Thangaraj K, Patterson N, et al. Reconstructing Indian population history. *Nature* 2009;461:489. doi: 10.1038/nature08365

41. Das S, Forer L, Schonherr S, et al. Next-generation genotype imputation service and methods. *Nat Genet* 2016;48(10):1284-87. doi: 10.1038/ng.3656

42. Iglesias AI, van der Lee SJ, Bonnemaijer PWM, et al. Haplotype reference consortium panel: Practical implications of imputations with large reference panels. *Hum Mutat* 2017;38(8):1025-32. doi: 10.1002/humu.23247

43. Loh P-R, Danecek P, Palamara PF, et al. Reference-based phasing using the Haplotype Reference Consortium panel. *Nat Genet* 2016;48:1443. doi: 10.1038/ng.3679

44. Wang K, Li M, Hadley D, et al. PennCNV: An integrated hidden Markov model designed for high-resolution copy number variation detection in whole-genome SNP genotyping data. *Genome Res* 2007;17(11):1665-74. doi: 10.1101/gr.6861907

45. Kendall KM, Rees E, Escott-Price V, et al. Cognitive Performance Among Carriers of Pathogenic Copy Number Variants: Analysis of 152,000 UK Biobank Subjects. *Biol Psychiatry* 2017;82(2):103-10. doi: <https://doi.org/10.1016/j.biopsych.2016.08.014>

46. Zuvich RL, Armstrong LL, Bielinski SJ, et al. Pitfalls of merging GWAS data: lessons learned in the eMERGE network and quality control procedures to maintain high data quality. *Genet Epidemiol* 2011;35(8):887-98. doi: 10.1002/gepi.20639

47. MacArthur JAL, Buniello A, Harris LW, et al. Workshop proceedings: GWAS summary statistics standards and sharing. *Cell Genomics* 2021;1(1):100004. doi: <https://doi.org/10.1016/j.xgen.2021.100004>

48. Choi SW, O'Reilly PF. PRSice-2: Polygenic Risk Score software for biobank-scale data. *GigaScience* 2019;8(7) doi: 10.1093/gigascience/giz082

49. Ge T, Chen C-Y, Ni Y, et al. Polygenic prediction via Bayesian regression and continuous shrinkage priors. *Nature Communications* 2019;10(1):1776. doi: 10.1038/s41467-019-09718-5

50. Bulik-Sullivan BK, Loh P-R, Finucane HK, et al. LD Score regression distinguishes confounding from polygenicity in genome-wide association studies. *Nat Genet* 2015;47(3):291-95. doi: 10.1038/ng.3211

<http://www.nature.com/ng/journal/v47/n3/abs/ng.3211.html#supplementary-information>
